# Imperforated cor triatriatum dexter in a dog with concurrent caudal vena cava wall mineralization

**DOI:** 10.1186/s13028-016-0269-5

**Published:** 2017-01-03

**Authors:** Tetyda Paulina Dobak, Gregory Starrak, Kathleen Linn, Elisabeth Christine Roberston Snead

**Affiliations:** 1 Department of Diagnostic Imaging, Faculty of Veterinary Medicine, Utrecht University, Yalelaan 108, 3508 TD Utrecht, The Netherlands; 2 Veterinary Medical Centre, Western College of Veterinary Medicine, University of Saskatchewan, 52 Campus Drive, Saskatoon, SK S7N 5B4 Canada

**Keywords:** Cor triatriatum, Congenital cardiac malformation, Budd-Chiari like syndrome, Vascular mineralization

## Abstract

**Background:**

Cor triatriatum dexter (CTD) is a rare congenital cardiac malformation with various manifestations and has been sporadically described in dogs. Clinically the dogs present with nonspecific signs of right heart failure or Budd-Chiari-like syndrome. Other associated concurrent cardiovascular anomalies are commonly reported. Diagnosis and full characterization of this complex malformation requires careful investigation and often a multimodal imaging approach.

**Case presentation:**

A 10-week-old, male intact, Golden Retriever was presented with clinical signs of stunted growth, anorexia, and progressive ascites. CTD imperforate with sole separation of the caudal vena cava (CdVC) and concurrent venous wall mineralization was conjointly diagnosed and fully characterized by echocardiography, non-selective angiography, computed tomography angiography and cardiac magnetic resonance imaging (MRI). This was successfully treated surgically and the dog returned to normal activity.

**Conclusion:**

To the author’s knowledge, this is the first case of CTD imperforate separating the CdVC from the right atrium (RA) with presumed secondary CdCV wall and hepatic parenchyma mineralization reported in a dog. CTD is an important and potentially correctable cause for the development of ascites in a young puppy. Accurate diagnosis of this complex cardiac anomaly is important for selection of the most appropriate curative treatment option.

## Background

Cor triatriatum dexter (CTD) has been infrequently reported in dogs and the overall prevalence is low [1]. With this cardiac anomaly, the embryonic right valve of the sinus venosus fails to regress, resulting in partitioning of the right atrium (RA) into two distinct chambers and effectively creating a triatrial heart [2]. The fibrous membrane dividing the RA is most commonly perforated, however, in some cases, like the one described, it can be imperforate leading to complete obstruction of the venous return to the heart from the caudal half of the body through the caudal vena cava (CdVC) and/or from the coronary sinus depending on its location.

Given the varying extent of regression failure seen in different cases, the venous return from the abdomen to the RA can be impeded to varying degrees. This may result in clinical signs suggestive of caudal right-sided congestive heart failure (CHF) or a Budd-Chiari-like syndrome [3].

Consequently, alternate venous pathways, including cavo-azygos shunting, are often observed in order to ensure venous return from the caudal extremities and abdomen back to the right atrium is possible.

Since CTD can present with a variety of concurrent cardiac anomalies including tricuspid valve dysplasia, dynamic subaortic stenosis, atrial septal defect (ASD), pulmonic valvular stenosis, persistent foramen ovale (PFO), ventricular septal defect, pericardial agenesis, persistent left cranial vena cava or Ebstein’s anomaly, which could impact outcome, it is important to carefully evaluate each case to determine if correction of the CTD will resolve the clinical signs and lead to a favourable outcome for the patient [4–6].

Diagnosis of CTD benefits from a multimodal diagnostic imaging approach in order to more precisely characterize this complex cardiovascular anomaly and to assist in determining the most appropriate treatment.

This report describes the characterization of a case of CTD in a puppy using various imaging modalities.

## Case presentation

A 10-week-old, male intact Golden Retriever (body weight 4.02 kg) was referred to the Veterinary Medical Centre of the Western College of Veterinary Medicine, Canada with clinical signs of stunted growth, anorexia, and a progressively distended abdomen. Previous thoracic and abdominal radiographs performed by the referring veterinarian revealed marked CdVC dilation and hepatomegaly. The dog was owned by a breeder and born to a clinically normal dam and sire. There were 10 puppies in the litter, the remainder of which were healthy based on assessment by the referring veterinarian at multiple assessment time points for routine vaccinations and deworming.

On physical examination, a pendulous and distended abdomen that was tense on palpation was appreciated. Despite being tachypnoeic and tachycardic, lung sounds were deemed normal on auscultation and there was no audible heart murmur, arrhythmia or jugular vein distension. No abnormalities were detected on a six lead electrocardiogram. The mucous membranes were pale but moist with a normal capillary refill time.

A complete blood count and serum biochemistry profile revealed a mild hypoalbuminemia (29 g/l) and a moderate microcytic hypochromic anaemia (Hct: 22%) with evidence of marked regeneration. Pre- and postprandial serum bile acids were normal.

**Fig. 1:**
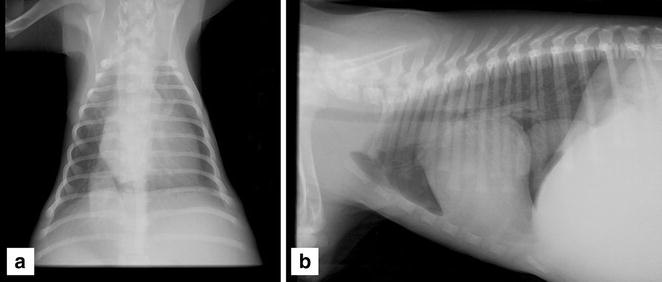
**a** Right lateral and **b** dorsoventral radiograph of the thorax. Note the tortuous, severely distended CdVC and bulge of the cardiac silhouette at the level of the RA

**Fig. 2:**
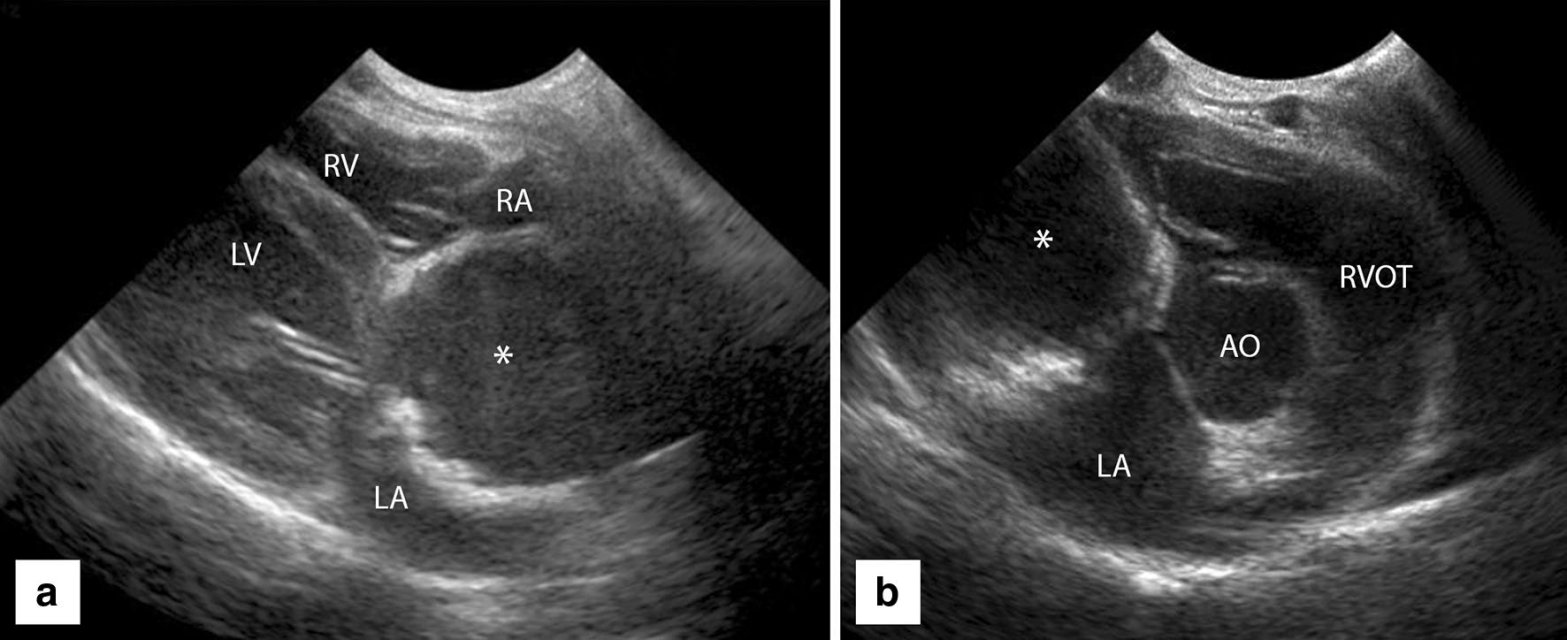
Echocardiography demonstrating **a** right long axis view and **b** right parasternal short axis view. **a** Large rounded thick walled structure (*asterisk*) at the cavoatrial junction is seen between both ‘normal’ atria

Repeat thoracic radiographs (Fig. 1) showed the presence of a severely distended and tortuous CdVC (CdVC/aortic ratio >1.5), kinking of the CdVC at the level of the cardiac base with a bulge of the cardiac silhouette at the level of the RA, and mild right heart enlargement. The pulmonary vasculature and airways were unremarkable. In the cranial abdomen, severe hepatomegaly and reduced abdominal serosal detail suggestive of ascites was noted. Abdominal ultrasound confirmed severe CdVC dilation, marked congestion of the hepatic veins, severe hepatomegaly and the presence of a small amount of free abdominal fluid. In addition, an incidental cholelith within the gall bladder was detected. Echocardiography showed a large spherical, thick-walled chamber filled with hypoechoic fluid at the level of the cavoatrial junction compressing both normal atria laterally in the right parasternal short axis view (Fig. 2).

**Fig. 3:**
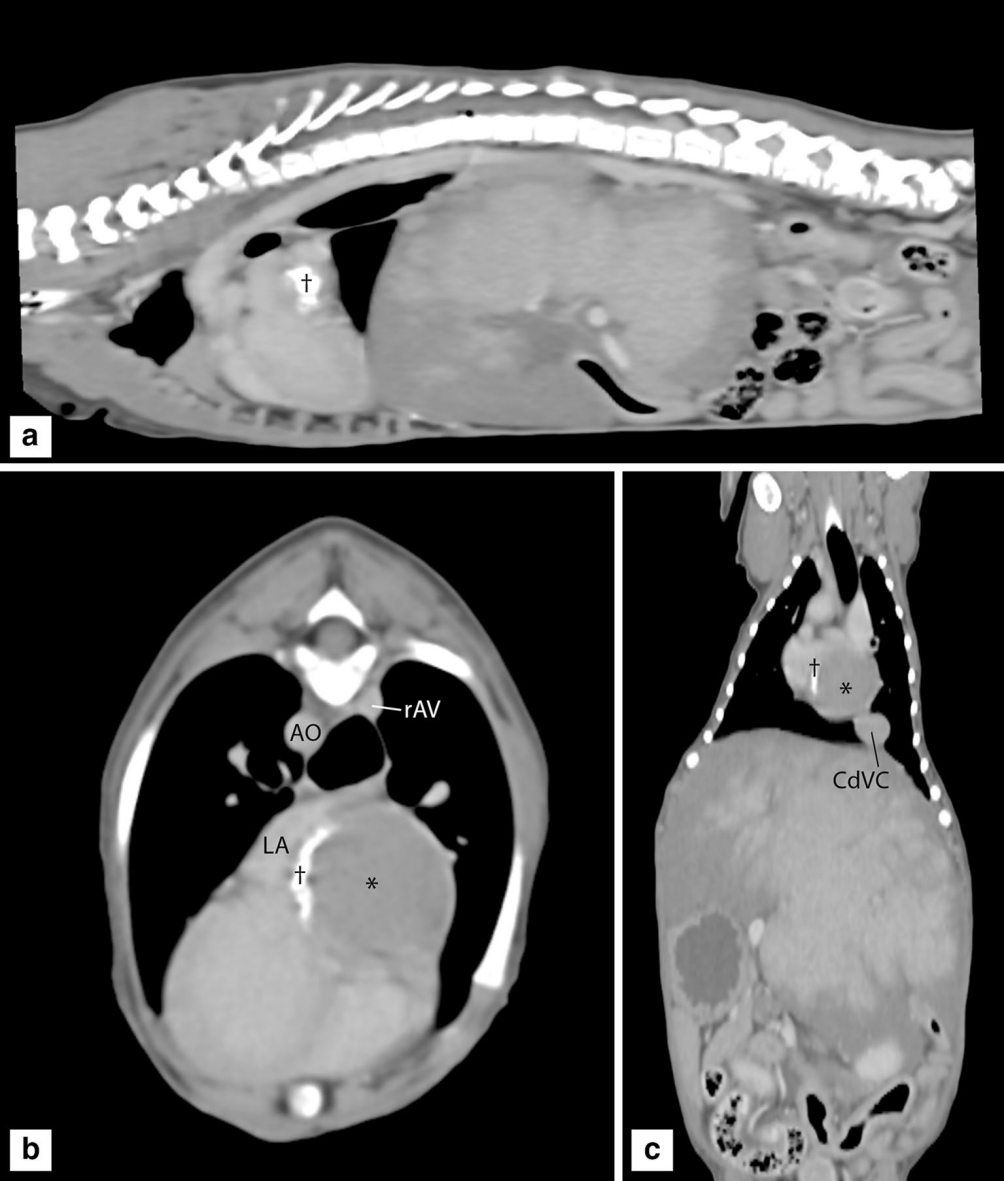
**a** Sagittal, **b** transverse and **c** dorsal images in a soft tissue window level 3 min post contrast injection. Fine mineral dense material (*dagger*) is seen within the wall between the dilated right atrial compartment (*asterisk*) and left atrium. Severe dilation of the CdVC, right azygos vein (rAV) and generalized hepatomegaly are noted

With Doppler-ultrasound no active flow was noted within this chamber, which was identified as the cranial extension of the CdVC. Based on these preliminary findings it was suspected that this dilation of the CdVC was walled-off completely from the ‘normal’ RA by a membrane at the cavo-atrial junction leading to a presumptive diagnosis of CTD with an imperforate atrial membrane. No other concurrent cardiac anomalies were appreciated. Contrast-enhanced echocardiography (‘bubble study’) was performed to help determine if there was any potential communication between either the cranial vena cava (CrVC) and the CdVC and the RA, respectively. When agitated saline was injected into the right cephalic vein, microbubbles were immediately identified in the RA confirming normal communication between the CrVC and the RA. However, when agitated saline was injected into the left lateral saphenous vein a lot of microbubbles were identified passing through the RA into the right ventricle despite only a few microbubbles seen reaching the distended cranial extent of CdVC at the level of the heart. In the mid abdominal CdVC, at the level of the diaphragm, a larger number of microbubbles were seen pooling and flowing retrograde on inhalation without sustained forward motion. This finding suggested that there was no communication between the CdVC and the RA and that the microbubbles had reached the right heart through an alternate venous pathway. A non-selective venous angiogram with Iohexol (Omnipaque^®^, 240 mg I/ml, dose 2 ml/kg) contrast injected into the left lateral saphenous vein confirmed obstruction of venous return to the heart through the CdVC with no contrast identified cranial to L2. Contrast was also identified within the distended right azygos vein, which was seen to empty directly into the RA. Computed tomography angiography (CTA) of the thorax and abdomen using a 16-slice helical CT scanner (Toshiba Aquilion 16, Toshiba Medical Systems) was performed under general anaesthesia. Images with 1 mm slice thickness were acquired before, immediately after and 1, 2 and 3 min after injection of Iohexol contrast into the left lateral saphenous vein. Axial compression of the right atrial chamber against the left atrium and a flat crescent-shaped tissue barrier between the CdVC and the left atrium in sagittal orientation was appreciated (Fig. 3).

The anomalous tissue was composed of a fine linear accumulation of irregular mineral dense material (±345 HU) over an area of approximately H 22 × L 16 mm. On ultrasound, this area had been visualized as hyperechoic tissue with distal acoustic shadowing consistent with mineralization. Following contrast injection, a markedly distended right azygos vein was seen from the level of T12 cranially to where it drained into the cranial part of the RA. Caudal to the heart the intrathoracic CdVC was markedly dilated measuring 14 mm in diameter and 30 × 23 mm at the junction with the RA (compared to the aortic diameter of 6.3 mm at the same level). The CdVC dilation extended across the diaphragm into the abdomen with marked dilation of the hepatic veins. Severe hepatomegaly with liver lobes extending caudal to the level of L5 was present along with persistent extensive contrast enhancement of the liver with sequential studies confirming obstruction of hepatic venous return to the heart and secondary hepatic congestion. Multifocal stippled mineralization of the hepatic parenchyma within the caudate liver lobe and the previously appreciated gall bladder cholelith were also apparent.

**Fig. 4:**
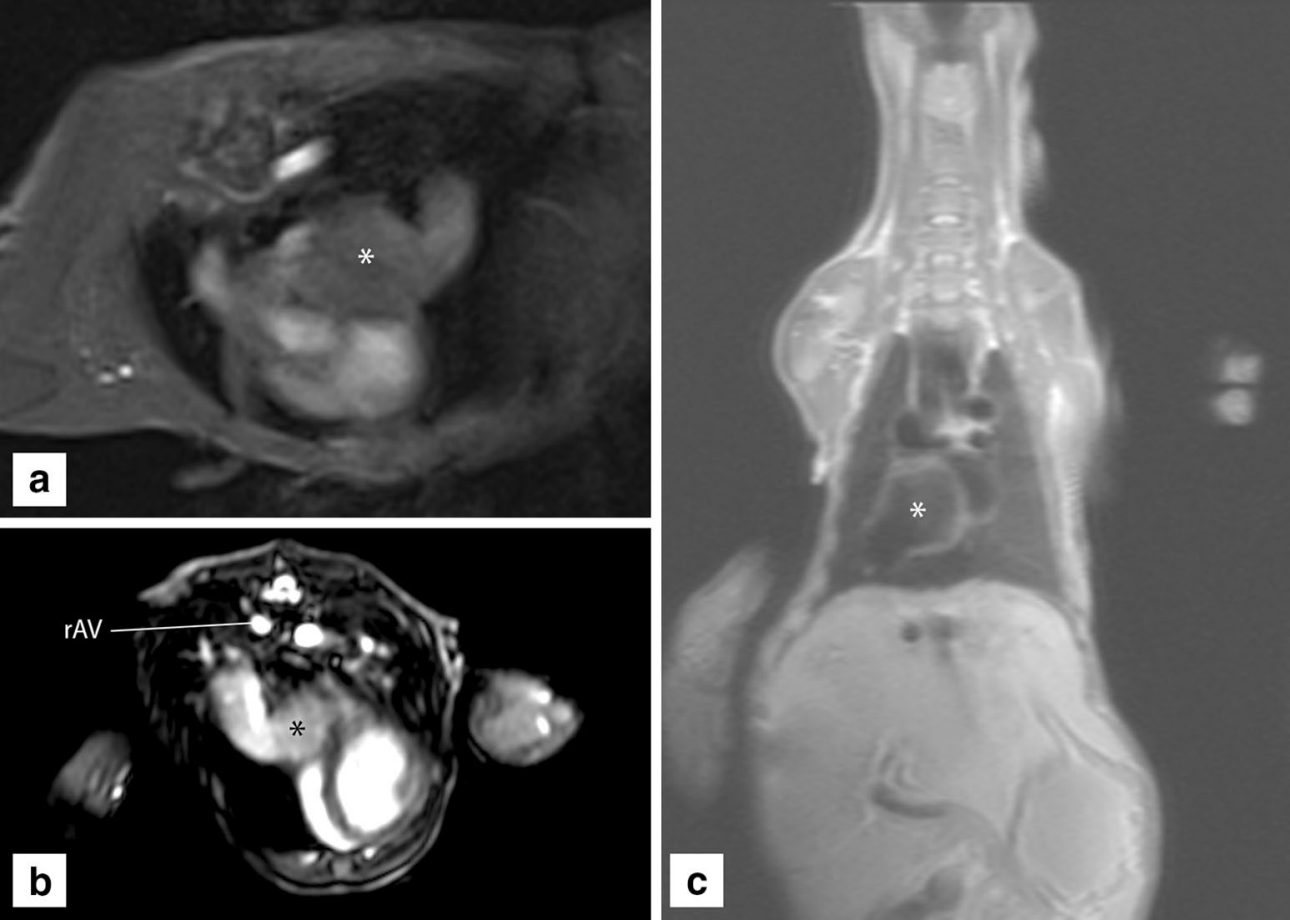
Cardiac MRI in **a** FLASH and **b** True FISP bright blood and **c** HASTE dark blood flow sequence. Note that no blood flow is seen within the cranial end of the CdVC (*asterisk*). Blood is flowing through the dilated right azygos vein (rAV)

A cardiac MRI study with a 1.5 T magnet (Symphony, Siemens) was also performed to permit further structural and functional assessment of the heart and major thoracic vasculature. The dog was positioned in sternal recumbency with two phased array coils around the thorax, one head coil cranial to the heart and an additional spine coil at the level of the heart. Cine sequences were retrospectively P-wave gated with a protocol for arrhythmia since ECG-gaiting could not be obtained due to magnetic interference. Images were acquired using a dark blood technique. For this purpose, T1 turbo spin echo (TSE) sequences (TE: 28 TR: 700) depicting the cardiac and vascular morphology, as well as T2 TSE (TE: 87 TR: 800) and HASTE (single shot) sequences (TE: 33 TR: 800, FOV 300, Matrix 106x 256, Slice thickness 5–6 mm) were obtained. For the purpose of visualization of the cardiac and vascular hemodynamic function during the cardiac cycle, 12 slice (True FISP, balanced sequence) Cine sequences were acquired in 2D volumes. Furthermore, a velocity encoded technique to demonstrate vascular flow (150 cm/s) through the sagittal plane was acquired with a FLASH (incoherent gradient echo-gradient spoiled) sequence (TE: 4.2 TR: 36). A thick membrane separating the spherical end of the CdVC from the cranial right atrial chamber and walling it off to the left atrium was confirmed (Fig. 4).

The blood flow from the CrVC, as well as from the distended right azygos vein, through the sinus venarum cavarum and into the cranial RA and into the right ventricle was unimpeded. Emptying of the coronary sinus into the cranial right atrial compartment also appeared normal. No vascular flow between the CdVC and RA was appreciated during all phases of the cardiac cycle again confirming the complete obstruction of normal caudal venous return to the RA.

**Fig. 5:**
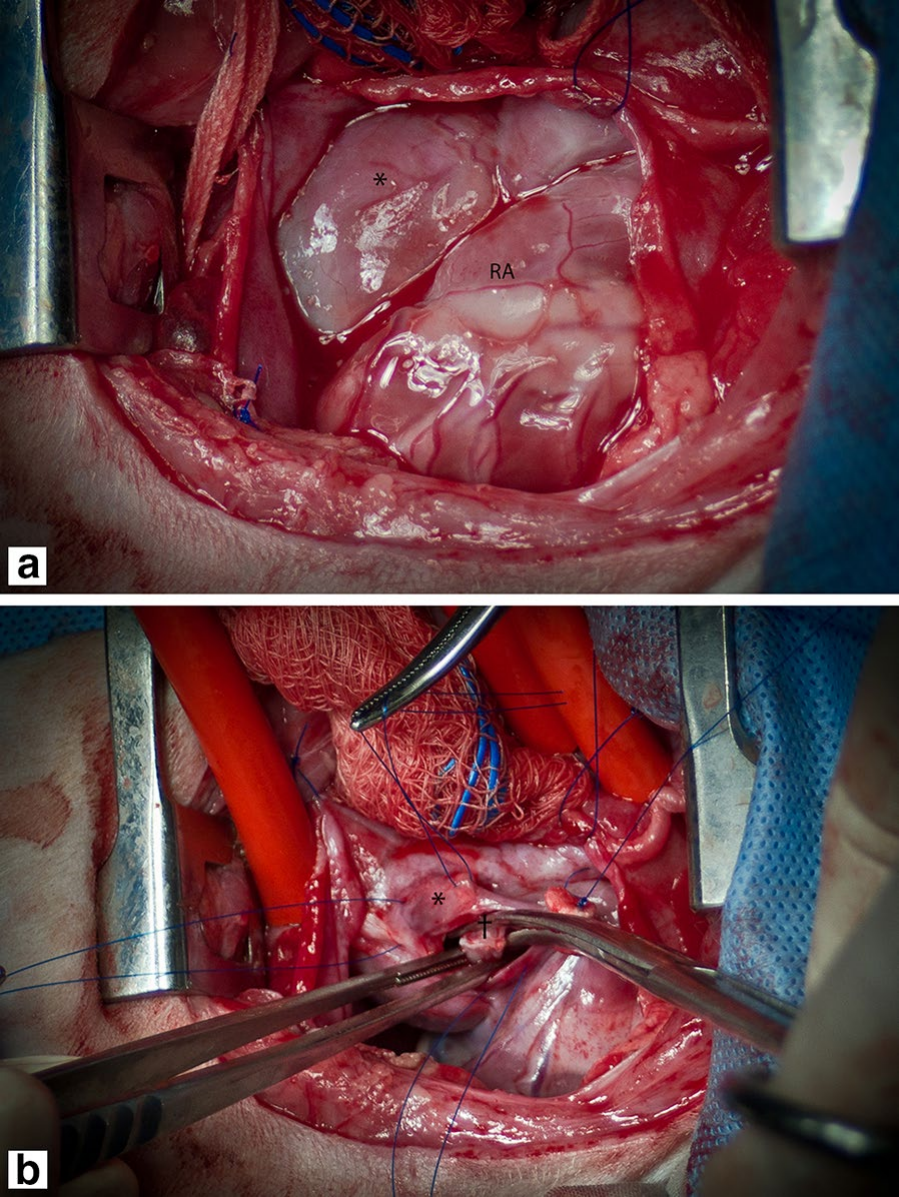
View through right lateral thoracotomy and pericardiotomy. **a** The markedly distended spherical cranial end of the caudal vena cava (*asterisk*) and right atrium (RA). **b** Right atriotomy under total inflow occlusion with partial excision of the anomalous membrane (*dagger*)

Human pediatric cardiologists consulted regarding this case advised that interventional treatment using balloon dilation or a cutting balloon was not a viable option so two weeks later surgical correction of the anomaly under total venous inflow occlusion with mild induced hypothermia was performed. The heart was accessed through a right 5th intercostal space thoracotomy and 100 ml of mildly cloudy pleural effusion were suctioned from the thoracic cavity. Intraoperatively the blind-ended pouch of the CdVC measured 4–5 cm in diameter and appeared continuous with the caudal wall of the cranial right atrial compartment (Fig. 5).

A normal intact thoracic duct could be identified dorsal to the CrVC. Following atriotomy, a part of the membrane separating the CdVC and RA was excised creating an opening of about 1–2 cm in diameter. The total inflow occlusion time was 2 min and the heart kept beating throughout. Immediately after restoration of the venous flow, marked reduction of CdVC dilation and improved filling of the RA was observed. Recovery from anaesthesia was uneventful. Postoperatively the dog was started on low-dose aspirin to reduce the risk of thrombus formation (5 mg/kg PO *q* 24 h for 7 days) and was discharged 5 days after surgery.

Postoperative follow-up echocardiography performed one week later revealed normal cardiac function with persistent patency of the membranostomy orifice, measuring 7.6 mm in diameter. Persistent mild distension of the CdVC where it entered the heart was appreciated, however, the distension of the remainder of the thoracic and abdominal portions of the CdVC had resolved. Normal filling of both atria was re-established and the pleural effusion had resolved but a small amount of peritoneal effusion around the liver lobes could still be appreciated. One month later, echocardiographic reassessment showed that the membranostomy orifice had remained patent measuring 10 mm in diameter. Cardiac function and CdVC size remained normal. After this the dog gradually returned to normal activity, growing fast and showing normal exercise tolerance. Over a period of more than 2 years follow-up the dog has continued to do well and has never shown any exercise intolerance during routine flyball and duck hunting activity.

## Conclusion

This case report provides a description of a rare congenital cardiac abnormality resulting in a triatrial heart partitioned by an imperforate membrane from the CdVC. Unique aspects of this case are the unusual location of the separating membrane and concurrent mineralization of the CdVC and hepatic parenchyma.

**Fig. 6:**
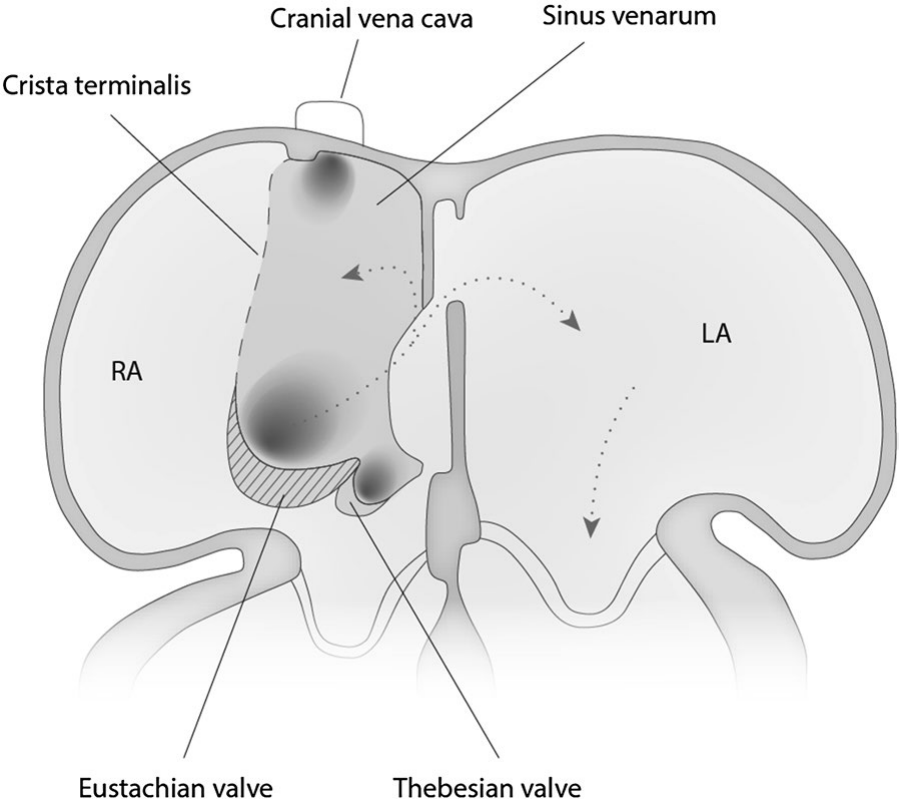
This figure demonstrates the flow of oxygenated blood from the sinus venosus into the left atrium (*arrows*) and components of the right sinus venosus valve after regression. Regression failure of the Eustachian valve (*hatched*), results in separation of the caudal vena cava from the right atrium (RA)

During embryological primary septation of the heart, the sinus venosus is formed by the distal part of the bent tube. The right horn of the sinus venosus gradually incorporates itself into the cranial part of the RA forming at a later stage the smooth sinus of the caval veins. The original embryologic RA forms the trabeculated caudal portion of the ultimate RA. The two portions of the RA are connected through a sinoatrial orifice, which initially is covered by the right and left venous valves on either side. The left venous valve ultimately becomes a part of the septum secundum while the right venous valve continues to divide the RA into two chambers and directs oxygenated blood entering the heart from the CdVC across the foramen ovale to the left atrium during foetal life. By 12 weeks post gestation in humans this sheet of tissue that represents the right sinus venosus valve normally regresses. After regression, the crista terminalis remains cranially and the Eustachian valve, the valve of the CdVC and the Thebesian valve, the valve of the coronary sinus, remain caudally (Fig. 6) [7].

Persistence of this right sinus venosus valve, which is the basis for the anomaly seen with classic CTD, results in separation of the RA into two compartments in the adult heart. We hypothesize that regression failure of only the caudal portion of the right sinus venosus valve, the Eustachian valve, that is the former valve of the CdVC, lead to the unique location of the imperforate membrane in our dog (Fig. 6).

In a triatrial heart, the two distinct compartments of the RA can be completely separated by an imperforate membrane or communicate through an orifice of variable size if the membrane is perforate. The hemodynamic consequences with CTD will vary with the degree of obstruction, e.g. complete vs. incomplete, and with the size of the opening if perforate [3]. With classic CTD the cranial RA has normal physiologic pressures and receives the venous drainage from the cranial half of the body from the CrVC. In the case described, the venous return from the CrVC, coronary sinus, and right azygos vein to the true RA were not impeded. If the membrane is perforated, high-pressured flow will also enter the true RA from the CdVC. However, the increased resistance impeding caudal venous return will lead to signs of caudal right-sided CHF (i.e., hepatic venous congestion, ascites) without evidence of cranial right-sided CHF signs (i.e., jugular vein distension). If the membrane is imperforated, like in this case, which is described less frequently than the perforated form [3, 8, 9], no communication exists between the two right atrial compartments divided by the anomalous membrane. Thrombotic or nonthrombotic obstruction of the hepatic venous outflow results in the classic triad of hepatomegaly, ascites, and abdominal pain secondary to development of postsinusoidal portal hypertension. For this reason, some authors term the resultant caudal right-sided CHF a Budd-Chiari-like syndrome. In our dog, the degree of hepatomegaly, dilation of the CdVC and the size of the anomalous caudal right atrial compartment was very profound compared to previously reported cases of imperforate CTD in dogs [3, 8–10].

Dogs with imperforate septa tend to form anomalous collateral vessels connecting the CdVC either directly to the right azygos vein or indirectly to the right azygos vein via the vertebral or intercostal venous circulation [8, 9, 11, 12]. In this case, indirect azygos continuation of the CdVC was found with suspected shunting through anastomosis with the vertebral venous circulation. Development of these collateral pathways permits some degree of decompression of the high-pressure caudal venous compartment. In some cases of CTD further decompression through the coronary sinus, or through concurrent PFO or ASD, which further function as effective pop-off valves, have been reported. Our dog lacked the additional latter possibilities for decompression. We speculate that the imperforate membrane with absence of additional ‘vents’ resulted in more extensive hemodynamic consequences resulting in severe postsinusoidal hypertension which could not be sufficiently compensated for by the collateral azygos continuation.

Computed tomography revealed the presence of multifocal mineral foci in the hepatic lobes in our dog. Degenerative mineralization secondary to severe hepatic congestion is a possible explanation. Hepatic bridging fibrosis and necrosis has been found on histopathological examination in two other cases of CTD [3, 13]. The cholelith formation in the gall bladder seen in such a young dog was suggestive of chronic cholestasis likely related to chronic hepatic congestion. Mineralization of the ascending aorta, coronary arteries, aortic and mitral valves as a result of chronic degenerative changes and pathological calcium phosphate deposition in dogs, are often of unknown etiology [14, 15]. We did not find a predisposing disease for the CdVC wall mineralization seen in our dog.

Echocardiographic examination in combination with angiography has established the diagnosis of CTD in many previous cases, although in some dogs cross-sectional imaging with other modalities has been required [6, 8, 16]. In the present case echocardiography allowed the detection of the congenital anomaly, however, CT and cardiac MRI facilitated a better understanding of the anatomy, relationship between anatomical structures, altered vascularization and the hemodynamic consequences of the anomaly with respect to the cardiovascular system. An accurate anatomic characterization of the anomaly and great vessels is essential for directing the most appropriate treatment option in these cases. Cardiac MRI has proven an excellent non-invasive diagnostic tool for the identification and characterization of congenital heart defects in man [17]. It has inherent advantages facilitating the morphological and hemodynamic assessment of the heart and major vasculature during stages of the cardiac cycle, allows for identification of concurrent extracardiac anomalies that might be present and provides better spatial resolution and superior tissue contrast compared with echocardiography. This has also reduced the requirement for radiographic angiography and the associated dangers for the subject and clinician [18]. To date cardiac MRI is rarely used in the veterinary field yet with developing technology previous limitations like size of the patient, acquisition time and fast heart rate (compared with man) can increasingly be overcome.

For a positive long-term outcome balloon dilatation [5, 8, 19, 20], including cutting balloon techniques [20] or surgical correction under inflow occlusion or extracorporal circulation [2, 4, 10, 21, 22] have been reported as the treatment of choice. In our dog, surgical membranostomy under inflow occlusion was successfully performed. The election of the surgical technique was based on the imperforate nature and the severe thickness of the membrane, which might have lead to complications with the balloon dilatation technique, as seen in previous reports [3, 19].

## Abbreviations

ASD: atrial septal defect
CdVC: caudal vena cava
CHF: congestive heart failure
CrVC: cranial vena cava
CT: computed tomography
CTA: computed tomography angiography
CTD: cor triatriatum dexter
ECG: electrocardiogram
FISP: fast imaging with steady-state free precession
FLASH: fast low angle shot
FOV: field of view
HASTE: half-Fourier acquisition single-shot turbo spin-echo
Hct: hematokrit
HU: hounsfield unit
MRI: magnet resonance imaging
PFO: persistent foramen ovale
PO: per os
RA: right atrium
rAV: right azygos vein
TE: echo time
TR: repetition time
TSE: turbo spin echo
